# Free cholesterol and cholesterol esters in bovine oocytes: Implications in survival and membrane raft organization after cryopreservation

**DOI:** 10.1371/journal.pone.0180451

**Published:** 2017-07-07

**Authors:** Jorgelina Buschiazzo, Glenda L. Ríos, Jesica R. Canizo, Silvia S. Antollini, Ricardo H. Alberio

**Affiliations:** 1 Biotecnología de la Reproducción, Departamento de Producción Animal, Instituto Nacional de Tecnología Agropecuaria (INTA), EEA Balcarce, Balcarce, Argentina; 2 Instituto de Investigaciones Bioquímicas de Bahía Blanca (CONICET-UNS), Camino La Carringanda, Bahía Blanca, Argentina; 3 Departamento de Biología, Bioquímica y Farmacia, Universidad Nacional del Sur (UNS), Bahía Blanca, Argentina; Universiteit Utrecht, NETHERLANDS

## Abstract

Part of the damage caused by cryopreservation of mammalian oocytes occurs at the plasma membrane. The addition of cholesterol to cell membranes as a strategy to make it more tolerant to cryopreservation has been little addressed in oocytes. In order to increase the survival of bovine oocytes after cryopreservation, we proposed not only to increase cholesterol level of oocyte membranes before vitrification but also to remove the added cholesterol after warming, thus recovering its original level. Results from our study showed that modulation of membrane cholesterol by methyl-β-cyclodextrin (MβCD) did not affect the apoptotic status of oocytes and improved viability after vitrification yielding levels of apoptosis closer to those of fresh oocytes. Fluorometric measurements based on an enzyme-coupled reaction that detects both free cholesterol (membrane) and cholesteryl esters (stored in lipid droplets), revealed that oocytes and cumulus cells present different levels of cholesterol depending on the seasonal period. Variations at membrane cholesterol level of oocytes were enough to account for the differences found in total cholesterol. Differences found in total cholesterol of cumulus cells were explained by the differences found in both the content of membrane cholesterol and of cholesterol esters. Cholesterol was incorporated into the oocyte plasma membrane as evidenced by comparative labeling of a fluorescent cholesterol. Oocytes and cumulus cells increased membrane cholesterol after incubation with MβCD/cholesterol and recovered their original level after cholesterol removal, regardless of the season. Finally, we evaluated the effect of vitrification on the putative raft molecule GM1. Cholesterol modulation also preserved membrane organization by maintaining ganglioside level at the plasma membrane. Results suggest a distinctive cholesterol metabolic status of cumulus-oocyte complexes (COCs) among seasons and a dynamic organizational structure of cholesterol homeostasis within the COC. Modulation of membrane cholesterol by MβCD improved survival of bovine oocytes and preserved integrity of GM1-related rafts after vitrification.

## Introduction

Long-term cryopreservation of female genetic material could be very useful for assisted reproductive technologies including breeding programs. However, obtaining viable embryos from cryopreserved oocytes has proved to be a difficult and inefficient labor in most mammals [1]. Vitrification is a cryopreservation procedure characterized by a high concentration of cryoprotectants and high cooling rate that mostly prevents the formation of ice crystals and replaces, at least for oocytes and embryos, the standard method of slow freezing. Unlike slow freezing, in which extracellular water crystallizes generating an osmotic gradient that dehydrate cells, in vitrification, extra and intracellular compartments vitrify after cellular dehydration [1]. Different vitrification devices have been designed in order to decrease the vitrified volume, thus increasing the cooling rate and reducing the exposure to cryoprotectants to minimize its toxic and osmotic hazardous effects (for review, see [1,2]). Still, in spite of the several advances in the field, the development of oocyte vitrification in cattle seems to have reached a *plateau* [3]. In line with this, although similar rates of survival and development have been achieved by different methods, the rates of development of bovine oocytes after vitrification are still low [3,4].

A major site of injury during mammalian oocyte cryopreservation is the plasma membrane. Part of the damage caused at the membrane level is induced by the change from a fluid state to an ordered state as temperature is reduced below the transition temperature of the membrane [5,6]. Graham and co-workers [7–11] prevented this damage by the addition of cholesterol to sperm membrane of different domestic animals in order to increase membrane fluidity at low temperatures and make it more tolerant to cryopreservation. To add cholesterol to the membrane and to increase its cholesterol level, they used methyl-β-cyclodextrin (MβCD), a cyclic oligosaccharide which has a high affinity for inclusion of cholesterol and other sterols in its hydrophobic cavity [12]. Modulation of plasma membrane cholesterol to increase post-cryopreservation survival is currently a booming topic for the male gamete of different species (for review, see [13]) but has been little addressed in oocytes. Previously, Horvath and Seidel [14] and, more recently, Spricigo et al. [15] reported the use of this method to add cholesterol to bovine oocytes prior to vitrification. Horvarth and Seidel [14] claimed that cholesterol could enter mature oocytes through the *cumulus* cells and the *zona pellucida*, improving its viability after vitrification in a chemically defined system. Spricigo et al. [15] improved nuclear maturation after treating immature bovine oocytes with cholesterol loaded MβCD prior to vitrification. However, these effects were not reflected in the production of embryos *in vitro* [14,15]. Whereas Horvath and Seidel [14] analyzed the presence and absence of serum in their assays, Spricigo et al. [15] used fetal calf serum in order to load MβCD with cholesterol from serum, and also during the recovery of *cumulus*-oocyte complexes (COCs), *in vitro* maturation and vitrification. More recently, rabbit oocytes with increased cholesterol content by exposure to MβCD/cholesterol complexes were found to fail to improve their developmental competence after either vitrification or slow freezing [16]. In none of these previous studies was the possibility of removing the added cholesterol analyzed after warming to recover membrane conditions prior to vitrification. Previous research has shown that whereas an excess of cholesterol does not affect meiotic progression, it induces mouse egg activation and it may cause female infertility [17]. Conversely, a sub-physiological level of cholesterol in mouse oocytes causes a delay in second polar body extrusion and low fertilization rates [18]. Cholesterol mediates membrane curvature during fusion events [19] and recent advances start to reveal common mechanisms also in gamete interaction [18,20,21]. It is clear that abnormal levels of membrane cholesterol could adversely affect fertilization and subsequent embryo development. Moreover, membrane cholesterol segregates laterally in small domains (10–200 nm) rich in sterols (cholesterol) and sphingolipids (sphingomyelin, gangliosides) known as membrane rafts [22]. Gangliosides are glycosphingolipids that contain sialic acid in their structure and, particularly, ganglioside GM1 has been found in the mouse oocyte and in the cleavage furrow of embryos [18,23–25]. These specialized microdomains permit plasma membrane sub-compartmentalization and formation of signaling platforms that mediate physiological responses. It has been shown that membrane raft integrity is necessary to efficiently accomplish fertilization in the mouse oocyte [18]. However, the importance of preserving membrane rafts in postcryopreserved bovine oocytes as well as in mammalian oocytes in general still remains unexplored. Exploring the effect of vitrification on membrane rafts may be particularly relevant considering the important role they play in cell signaling and gamete interaction.

In order to increase the survival of bovine oocytes after cryopreservation, we proposed not only to increase cholesterol level of oocyte membranes before vitrification but also to remove the added cholesterol after warming to recover its original level. In this respect, MβCD is a unique tool to modulate membrane cholesterol in living cells. The high affinity of MβCD for cholesterol can be used not only to generate cholesterol inclusion complexes that donate cholesterol to the membrane but also to remove cholesterol from biological membranes when used free of cholesterol [12]. The objectives of this study were to: (1) modulate cholesterol from membranes of bovine oocytes during vitrification, (2) determine the time of exposure to MβCD at which oocyte viability is not compromised, (3) quantify cholesterol incorporation by oocytes and *cumulus* cells, and (4) evaluate possible disturbance of membrane organization caused by cryopreservation through the analysis of the raft marker GM1 in living oocytes. Further understanding how bovine oocytes modulate cholesterol under these experimental conditions is a starting point to increase cryotolerance and to improve developmental competence of vitrified-warmed oocytes.

## Materials and methods

### 1- Chemicals and reagents

All chemicals and reagents were purchased from Sigma-Aldrich (St. Louis, MO, USA) unless otherwise stated.

### 2- Oocyte collection and *in vitro* maturation

#### 2.1 Oocyte collection

Bovine ovaries from cycling beef heifers (*Bos taurus*) were collected from local slaughterhouses and transported within 60–90 minutes in a thermic container to the laboratory. COCs were aspirated from follicles ranging from 2 to 8 mm in diameter by a vacuum system. COCs with homogeneous ooplasm and more than four complete layers of *cumulus* cells, corresponding to grades 1 and 2 according to de Loos et al. [26], were selected under a stereomicroscope and washed 3 times in phenol red-free HEPES buffered Synthetic Oviductal Fluid (H-SOF) supplemented with 1% polyvinyl alcohol (PVA) (w:v).

#### 2.2 *In vitro* maturation

Selected COCs were incubated in four-well culture plates (NUNC, Thermo Fisher Scientific, Loughborough, Leicestershire, UK) in groups of 60 per well, with 400 μl of serum- and gonadotropin-free maturation medium: M199 with 0.1 mg/ml L-glutamine and 2.2 mg/ml NaHCO_3_ supplemented with 10 ng/ml epidermal growth factor (EGF) [27,28], 30 μg/ml hyaluronic acid, and 100 μM cysteamine as anti-oxidant. COCs were incubated for 20 hours at 38.5°C under 5% CO_2_ in humidified air. When necessary, *cumulus* cells were removed either mechanically or by a brief exposure to 1 mg/ml hyaluronidase (Type IV-S from bovine testes), and *zona pellucida* (ZP) was dissolved with 1 mg/ml pronase (protease from *Streptomyces griseus*) under visual monitoring. ZP-free oocytes were rapidly washed 5 times and kept at 38.5°C under 5% CO_2_ in humidified air for 1-hour recovery. Experimental groups were defined as COCs matured in chemically defined medium, namely (1) without treatment (control), (2) loaded with cholesterol the last 45 minutes or 2 hours of maturation, (3) loaded with cholesterol the last 45 minutes or 2 hours of maturation and subsequently depleted of cholesterol for the same periods of time.

### 3- Cholesterol enrichment and depletion with methyl-β-cyclodextrin

MβCD complexed with cholesterol or alone was used to enrich and remove cholesterol from cell membranes, respectively. A stock solution of MβCD (0.5 M) in M199 was stored at 4°C in a glass tube. MβCD/cholesterol complexes (molar ratio 8:1) were prepared in M199 according to Christian et al. [29]. Briefly, cholesterol in chloroform:methanol 1:1 (v:v) was completely dried under a stream of nitrogen. A 15 mM MβCD aqueous solution was subsequently added to the dried material. The mixture was clarified by vigorous mixing, sonicated in bath sonication for 1–3 minutes and incubated in a rotating water bath at 37°C overnight. Before using the solution, it was filtered through a 0.45 μm syringe filter and equilibrated at the incubator.

COCs with partially removed *cumulus* cells (2–3 *cumulus* cell layers) and the bulk of *cumulus* cells were incubated separately with 15 mM MβCD/cholesterol for 45 minutes or 2 hours at 38.5°C under 5% CO_2_ in humidified air during the last period of *in vitro* maturation. In addition, in order to remove the added cholesterol from membranes, COCs were incubated with different concentrations of “empty” MβCD for 45 minutes or 2 hours. Before incubation with fluorescent probe Amplex® Red, oocytes were completely denuded.

### 4- Vitrification and warming

COCs with partially removed *cumulus* cells (2–3 *cumulus* cell layers) were vitrified with the surface device Cryotech® (ex Cryotop®) using vitrification and warming solutions of known composition. These protein free solutions were adapted from the literature available [3,30] replacing serum by the synthetic polymer PVA. Phenol red- and calcium-free H-SOF supplemented with 1% PVA was used to handle COCs and as base medium to prepare vitrification and warming solutions [30]. Vitrification was performed following the protocol of Zhou et al. [3] with some modifications. Briefly, COCs with 2–3 *cumulus* cell layers were vitrified with heated stage at 39°C (except for cholesterol loaded COCs that were vitrified at room temperature) and vitrification solutions at room temperature (25–27°C). COCs were equilibrated for 10 minutes in Equilibration Solution (ES) with 7.5% ethylene glycol and 7.5% DMSO and vitrified in Vitrification Solution (VS) with 15% ethylene glycol, 15% DMSO and 0.5M sucrose for 45–60 seconds including mounting onto Cryotech® (3 COCs/Cryotech®) and plunging into liquid nitrogen. Almost all VS was removed to leave only a thin layer covering the COCs. Warming was performed stepwise into decreasing sucrose solutions (1 M for 1 minute and 0.5 M for 3 minutes) and subsequently washed twice for 5 minutes in H-SOF/PVA. The heated stage was used at 39°C and warming solutions were maintained at 37°C during the whole procedure. Recovery of COCs was achieved at the incubator for 2 hours.

### 5- *In situ* detection of activated caspases

Apoptosis was analyzed in living cells through detection of activated caspases with a specific fluorescent inhibitor (VAD-FMK-FITC, Calbiochem®). Caspase inhibitor (VAD-FMK) conjugated to FITC is cell permeable, nontoxic and irreversibly binds to activated caspases in apoptotic cells. Denuded oocytes were incubated in 1:300 VAD-FMK-FITC in M199 for 45 minutes at 38.5°C under 5% CO_2_ in humidified air. Oocytes were washed in H-SOF/PVA with 1:500 propidium iodide (Calbiochem®) to assess membrane integrity. They were finally mounted for detection by fluorescence microscopy using a B-2A filter (microscope Nikon TE-300; Nikon, Tokyo, Japan). Oocytes showing brilliant green fluorescence were considered caspase positive.

### 6- Quantification of free cholesterol and cholesterol esters by Amplex® Red

Based on an enzyme-coupled reaction that detects both free cholesterol and cholesteryl esters, cholesterol levels were measured through a fluorometric method (Amplex® Red Cholesterol Assay Kit, Molecular Probes®) using an SLM model 4800 spectrofluorimeter (SLM Instruments, Urbana, IL) with a vertically polarized light beam from a Hannovia 200-W Hg/Xe arc lamp, obtained by a Glan-Thompson polarizer (4-nm excitation and emission slits), and 3- x 3-mm quartz cuvettes. Temperature for the assay (37°C) was set with a thermostat-controlled circulating bath (Haake, Darmstadt, Germany). Free cholesterol was measured performing incubation for 30 minutes in the dark. The fraction of cholesterol that is in the form of cholesterol esters was determined adding cholesterol esterase after free cholesterol measurement (time zero) and recording measurements after 30, 60 and 90 minutes. The latter incubation-time was the maximum at which all measurements reached a *plateau*. Measurements were performed in samples of 45–50 matured oocytes and the corresponding *cumulus* cells derived from MβCD-treated or untreated COCs. Fluorescence was measured with an excitation of 560 nm and emission of 590 nm. Background fluorescence determined for the non-cholesterol control sample was subtracted from each value. Cholesterol content was expressed as pmol of cholesterol per oocyte and nmol of cholesterol per mg of protein for *cumulus* cells. Protein content of *cumulus* cells was quantified by a direct method using a spectrophotometer (Picodrop, P100, UK).

### 7- Live cell imaging of lipids

#### 7.1 Staining of lipid droplets with Nile Red

Denuded oocytes were incubated with 1 μg/ml Nile Red (Molecular Probes®) in H-SOF/PVA for 5 minutes at room temperature. The fluorescent lipophilic dye was excited by a 450 to 500 nm line and yellow emission was detected with a B-2A filter. Digital photographs of the equatorial part of the oocyte were taken with a 20X objective in an epifluorescence inverted microscope (Eclipse TE-300; Nikon, Tokyo, Japan) connected to a DS-Fi1c camera (Nikon, Tokyo, Japan). Fluorescence intensity from oocytes was measured using ImageJ (Fiji) software [31]. Barceló-Fimbres and Seidel [32] validated the use of Nile Red to quantify the content of cytoplasmic neutral lipids in bovine oocytes and embryos.

#### 7.2 Fluorescent cholesterol in living oocytes

A stock solution (5 mM) of the fluorescent cholesterol analog, BODIPY-cholesterol (BPY-chol; Avanti Polar Lipids), was prepared in ethanol and stored in a dark glass tube under nitrogen at -20°C. Working solution (1 μM) was obtained diluting the stock in M199 medium (amount of ethanol less than 1%). Pulse-labeling was performed incubating COCs, *cumulus*-free oocytes and ZP-free oocytes with BPY-chol for 5 minutes at 38.5°C. Oocytes were immediately washed and subsequently imaged to avoid internalization of the lipid probe. Digital photographs were taken with a 20X objective in an epifluorescence inverted microscope (Eclipse TE-300; Nikon, Tokyo, Japan) connected to a DS-Fi1c camera (Nikon, Tokyo, Japan). Quantification of fluorescence intensity at the plasma membrane was measured in ZP-free oocytes by outlining regions of interest (ROI) in background corrected images using ImageJ (Fiji) [31].

#### 7.3 Localization of the raft marker lipid GM1

Glycosphingolipid GM1 was detected in living *cumulus*-free oocytes by using the fluorescent-labeled cholera toxin B subunit (CTB-AF^488^, Molecular Probes®), which binds specifically to the ganglioside. Oocytes were incubated at 38.5°C for 10 minutes in M199 supplemented with CTB-AF^488^ (20 μg/ml), washed with H-SOF/PVA, mounted and immediately imaged in an epifluorescence inverted microscope (Eclipse TE-300; DS-Fi1c camera, Nikon, Tokyo, Japan) to avoid internalization of toxin-GM1. Quantification of fluorescence intensity at the plasma membrane was measured by outlining regions of interest (ROI) in background corrected images using ImageJ (Fiji) [31].

### 8- Statistical analysis

Statistical analysis was carried out using InfoStat software [33]. Fluorescence intensity was analyzed through Student’s t test when two mean values were compared and analysis of variance (ANOVA) when more than two mean values were compared, followed by *post hoc* test analysis of multiple comparisons Bonferroni or Fisher’s Least Significant Difference (LSD Fisher). Caspase variable (binomial distribution) was compared using Generalized Linear Mixed Models (GLMM) with Binomial family, logit link function and LSD Fisher contrast. Cholesterol content was analyzed through ANOVA and *post hoc* tests of Bonferroni for autumn-spring samples and LSD Fisher for winter-summer samples. Differences were considered significant at *P*<0.05.

## Results

### Effect of cholesterol incorporation and depletion on oocyte viability

Cholesterol-binding drug MβCD was used in order to increase membrane cholesterol content before vitrification and to remove the added cholesterol after warming. COCs with partially removed *cumulus* cells were incubated with 15 mM MβCD/cholesterol for either 45 minutes or 2 hours during the last period of *in vitro* maturation. Matured COCs were subsequently vitrified-warmed and depleted of cholesterol within the period of recovery at the incubator. COCs loaded with cholesterol for 45 minutes were depleted of cholesterol for 45 minutes with 4.25 mM MβCD after warming and maintained in M199 at the incubator until completing 2 hours. COCs loaded with cholesterol for 2 hours were depleted of cholesterol with 8.5 mM MβCD for the same period of time.

**Fig 1:**
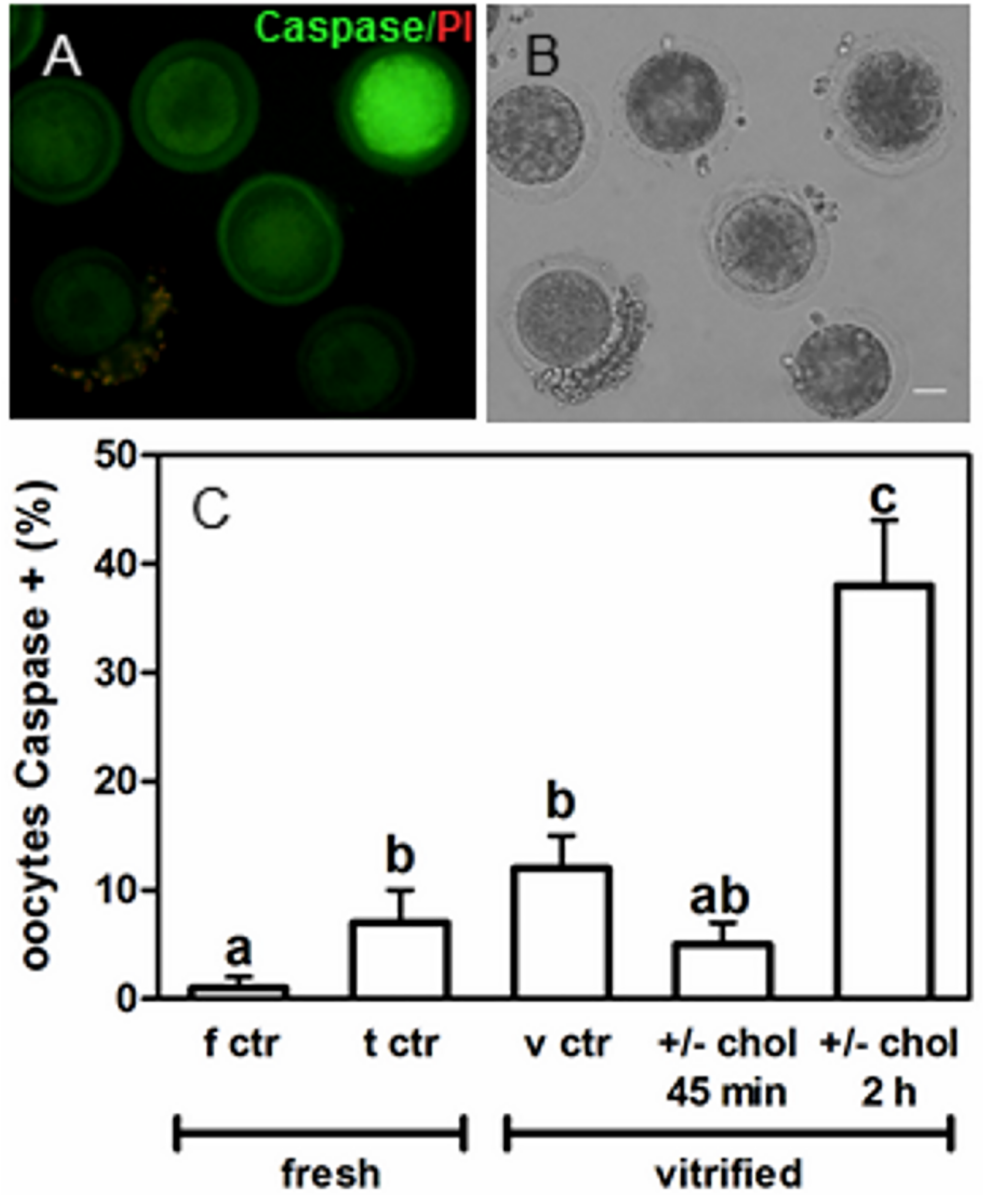
Effect of the exposure to cryoprotectans, vitrification, and cholesterol modulation during vitrification, on oocyte viability. (A) Partially denuded oocytes were incubated in VAD-FMK-FITC to detect *in situ* activated caspases and subsequently washed in the presence of propidium iodide to assess membrane integrity. Oocytes showing brilliant green fluorescence were considered caspase positive and *cumulus* cells showing green/orange fluorescence were considered positive for either caspases or both markers. (B) Bright field, scale bar: 25 μm. (C) Percentage of oocytes caspase positive in the fresh control group (f ctr), in the toxicity control group (exposed to vitrification and warming solutions; t ctr), in the vitrified control group (v ctr) and in treatment groups in which cholesterol was added to oocytes prior to vitrification and removed after warming (+/-chol) for periods of 45 minutes and 2 hours for both processes (enrichment-depletion). Data represent the mean ± SEM of 8 independent experiments with ~20 COCs for each condition. Caspase variable was compared using Mix Lineal Generalized Models (MLGM) with Binomial family, logit link and LSD Fisher contrast. Different letters (a-c) denote significant differences (*P*<0.05).

To ensure that the treatment with MβCD produced no alterations in the membrane with negative implications in viability, programmed cell death (apoptosis) indicator was analyzed. Detection of activated caspases was analyzed in live cells by a fluorescent specific inhibitor (FITC-VAD-FMK). This permeable fluorescent probe binds irreversibly to activated caspases in apoptotic cells allowing direct detection by fluorescence microscopy (Fig 1A). Moreover, these assays were carried out in the presence of propidium iodide to assess possible alterations in membrane integrity. This method allowed the analysis of the possible effect of modulating cholesterol (enrichment/removal) in vitrification as well as the effect of vitrification itself and the toxicity caused by exposure to vitrification and warming solutions. Results revealed that vitrification generated 12% of oocytes with activated caspases while fresh oocytes presented only ~1% of oocytes caspase positive (Fig 1C). In addition, when cholesterol was incorporated into oocytes for 2 hours prior to vitrification and removed after warming, the percentage of caspase activation was 26% higher than that in the vitrified control (12%) (Fig 1C). No statistical difference in caspase activation was found when cholesterol was incorporated into oocytes for 45 minutes before vitrification and removed after warming, with respect to the vitrified control. Moreover, no statistical difference was found in caspase activation compared to the fresh control. Interestingly, cholesterol modulation of oocytes for 45 minutes showed a similar level of caspase positive oocytes with respect to toxicity control (5% vs 7%, respectively) in which untreated oocytes were exposed to vitrification and warming solutions with no vitrification. In addition, oocytes were not positive for propidium iodide (except for some isolated case) in any of the experimental groups analyzed. On the contrary, when *cumulus* cells were left during incubation with fluorescent probes, they showed caspase/propidium iodide positive staining (Fig 1A) at both incubation times.

### Estimation of relative cholesterol incorporation into bovine oocytes

**Fig 2:**
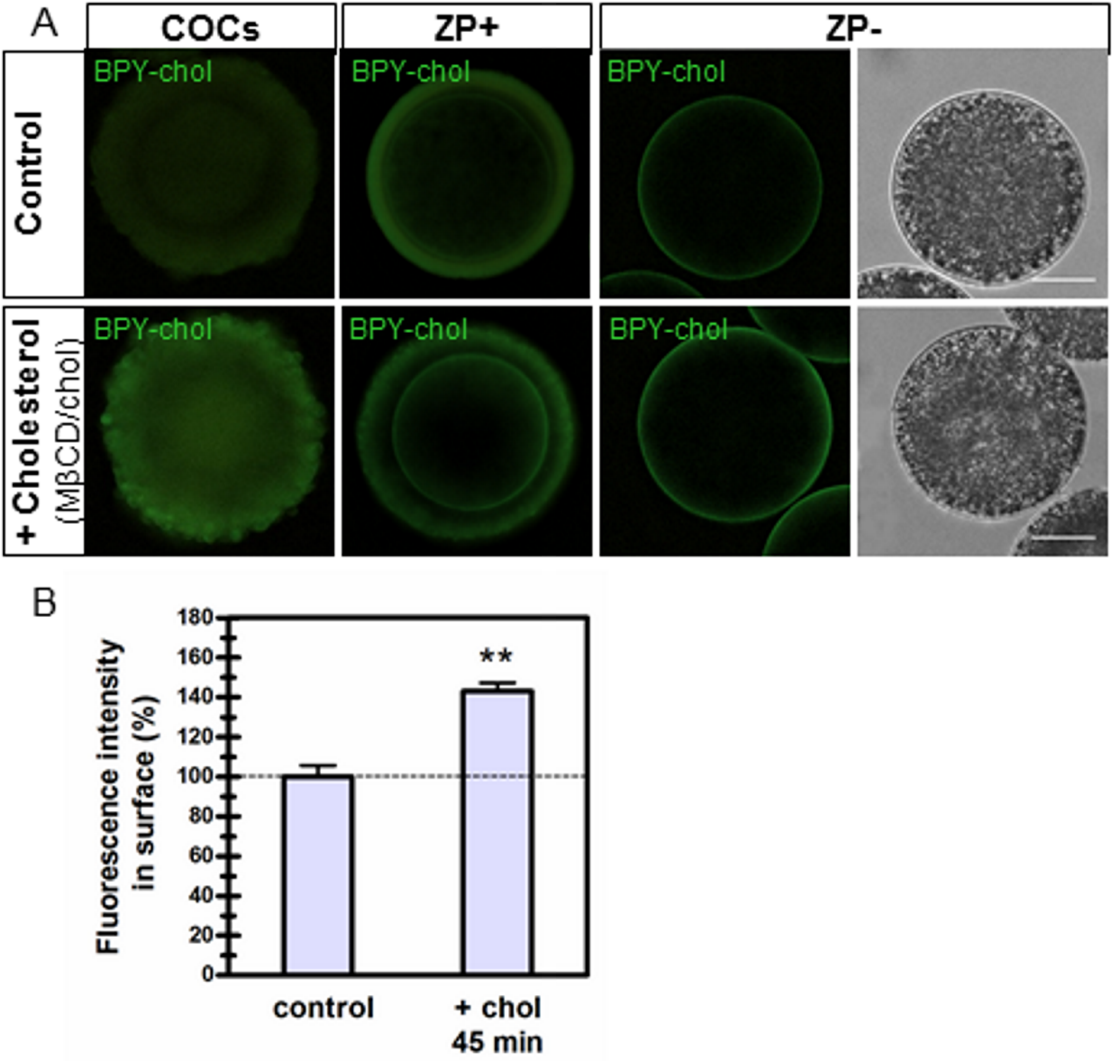
Incorporation of cholesterol into *cumulus*-oocyte complexes estimated by BODIPY-cholesterol labeling in living cells. (A) *Cumulus*-oocyte complexes (COCs), *cumulus*-free oocytes (ZP+) and ZP-free oocytes (ZP-) were incubated with 1 μM BODIPY-cholesterol after MβCD/cholesterol treatment of COCs for 45 minutes (lower panels). Upper panels show control conditions. Right panels show bright field; scale bar: 25μm. (B) Fluorescence intensity in ZP-free oocytes quantified with *Image J* software. Bars represent the mean ± SEM of 3 replicates from a total of 25 control oocytes and 28 cholesterol-loaded oocytes. Comparison of mean values was performed using Student *t* test. Asterisks denote significant differences (*P*<0.01). BPY-chol: BODIPY-cholesterol; chol: cholesterol; MβCD: methyl-β-cyclodextrin.

Taking into account that modulation of cholesterol for 45 minutes did not affect the apoptotic status of oocyte post-vitrification, fluorescent cholesterol analog BPY-chol was used to estimate the extent of cholesterol incorporation under these experimental conditions. COCs, *cumulus*-free oocytes and ZP-free oocytes were incubated with 1 μM BPY-chol after MβCD/cholesterol treatment of COCs for 45 minutes (Fig 2A). Incubation was performed until finding a time-point (5 minutes) at which the fluorescent cholesterol was highly located at the plasma membrane of the oocyte. That cholesterol incorporation mediated by MβCD/cholesterol complexes occurred effectively as well as its extent were both rapidly verified. An increase in fluorescence intensity was observed under all conditions analyzed (Fig 2A, lower panels) but quantification of fluorescence was performed in ZP-free oocytes (Fig 2B). Cholesterol-specific fluorescence in the plasma membrane increased ~43% after MβCD/cholesterol treatment compared to BPY-chol-control oocytes (Fig 2B).

### Response of bovine oocytes to cholesterol removal

**Fig 3:**
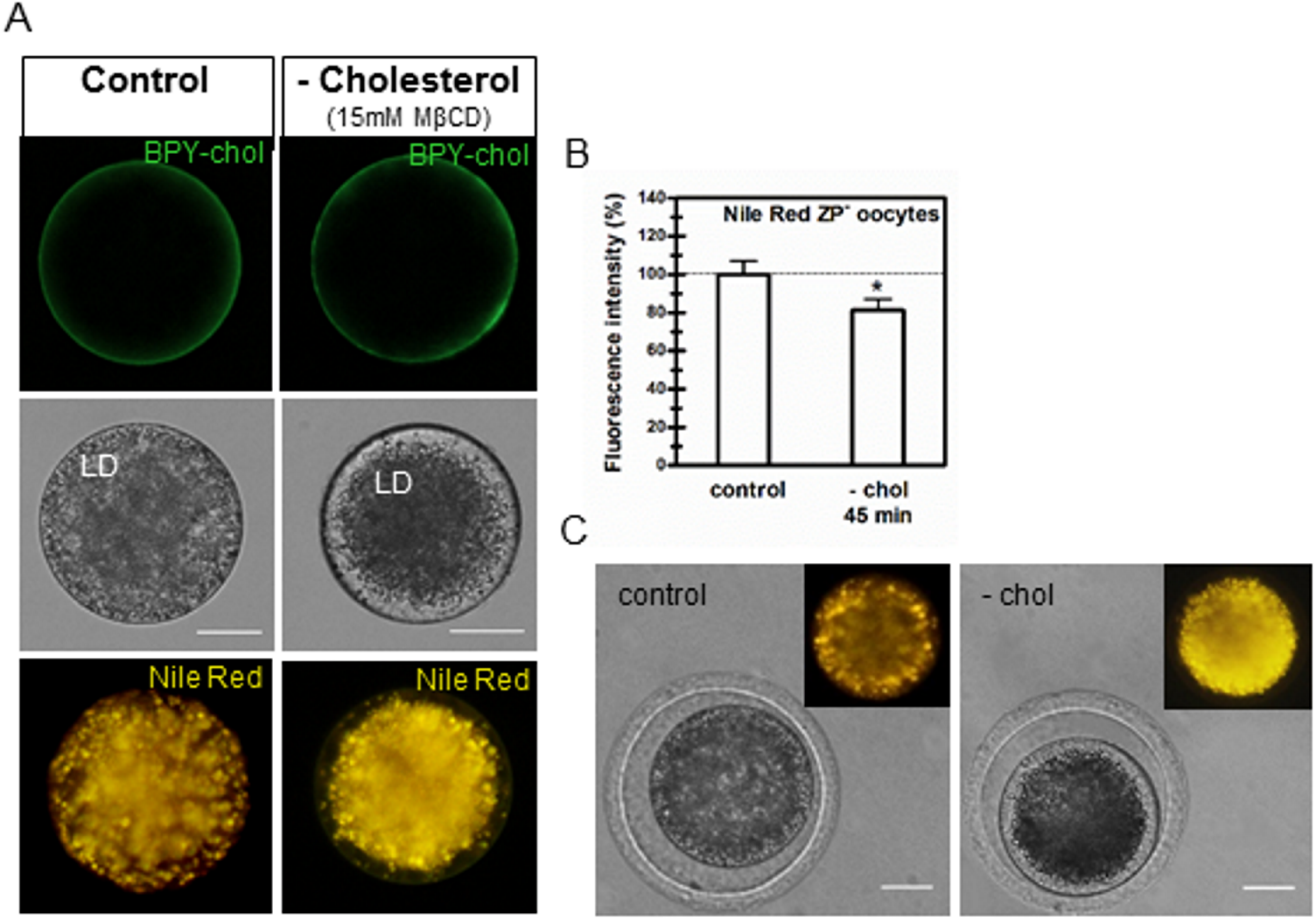
Effect of MβCD treatment on ZP-free and ZP-intact bovine oocytes. (A) BODIPY-cholesterol fluorescence in ZP-free oocytes directly exposed or not to 15mM MβCD for 45 minutes (upper panels). Bright field showing the distribution of lipid droplets (LD), scale bar: 25 μm (middle panels). Nile Red staining of oocyte lipid droplets (lower panels). Images are representative of 28 control oocytes and 29 MβCD-treated oocytes for BODIPY-cholesterol, and 28 control oocytes and 32 MβCD-treated oocytes for Nile Red. (B) Fluorescence intensity of Nile Red in ZP-free oocytes quantified with *Image J* software. Comparison of mean values was performed using Student *t* test. Asterisk denote significant differences (*P*<0.05). (C) Bright field showing the distribution of LD in ZP-intact oocytes exposed or not to 15mM MβCD for 45 minutes. Control oocytes showed the same pattern (40/40) while 16/45 oocytes showed the cortex devoid of LD in the MβCD-treated group. Scale bar: 25 μm. Inserts show Nile Red staining. BPY-chol: BODIPY-cholesterol; chol: cholesterol; MβCD: methyl-β-cyclodextrin.

Once incubation times with MβCD/cholesterol (45 minutes) and BPY-chol (5 minutes) were determined, we analyzed the response of oocytes to cholesterol depletion with no previous cholesterol loading or vitrification. Interestingly, ZP-intact oocytes incubated with 15 mM “empty” MβCD for 45 minutes showed effects only at lipid droplet level (Fig 3C). In order to simplify the contribution of any other unknown variable and to effectively assess cholesterol removal, ZP-free oocytes were directly exposed to 15 mM MβCD. Even under this direct exposure, treated oocytes showed no difference in membrane BPY chol fluorescence level compared to controls (Fig 3A, upper panels). Conversely, MβCD-treated oocytes showed marked differences in the distribution of lipid droplets (LD) observed in transmission images (Fig 3A, middle panels). The cortex of MβCD-treated oocytes was devoid of LD compared to untreated oocytes. Fluorescent probe Nile Red was in fact used to visualize these differences in the distribution of LD in MβCD-treated oocytes. Nile Red is a lipophilic probe that fluoresces yellow with neutral lipids stored in LD, such as triacylglycerols and cholesterol esters. Absence of LD in the oocyte cortex was confirmed by specific staining with Nile Red in live oocytes (Fig 3A, lower panels). Quantification of Nile Red fluorescence intensity showed a decrease of the emitted fluorescence in ZP-free oocytes of the MβCD-treated group (Fig 3B). When ZP-intact oocytes were exposed to MβCD under the same conditions, this distribution pattern of LD was observed in 35.5 ± 4.2% of the oocytes, with the cortex showing a lesser extent of absence of LD (Fig 3C).

### Quantification of free cholesterol and cholesterol esters in bovine oocytes and *Cumulus* cells

**Fig 4:**
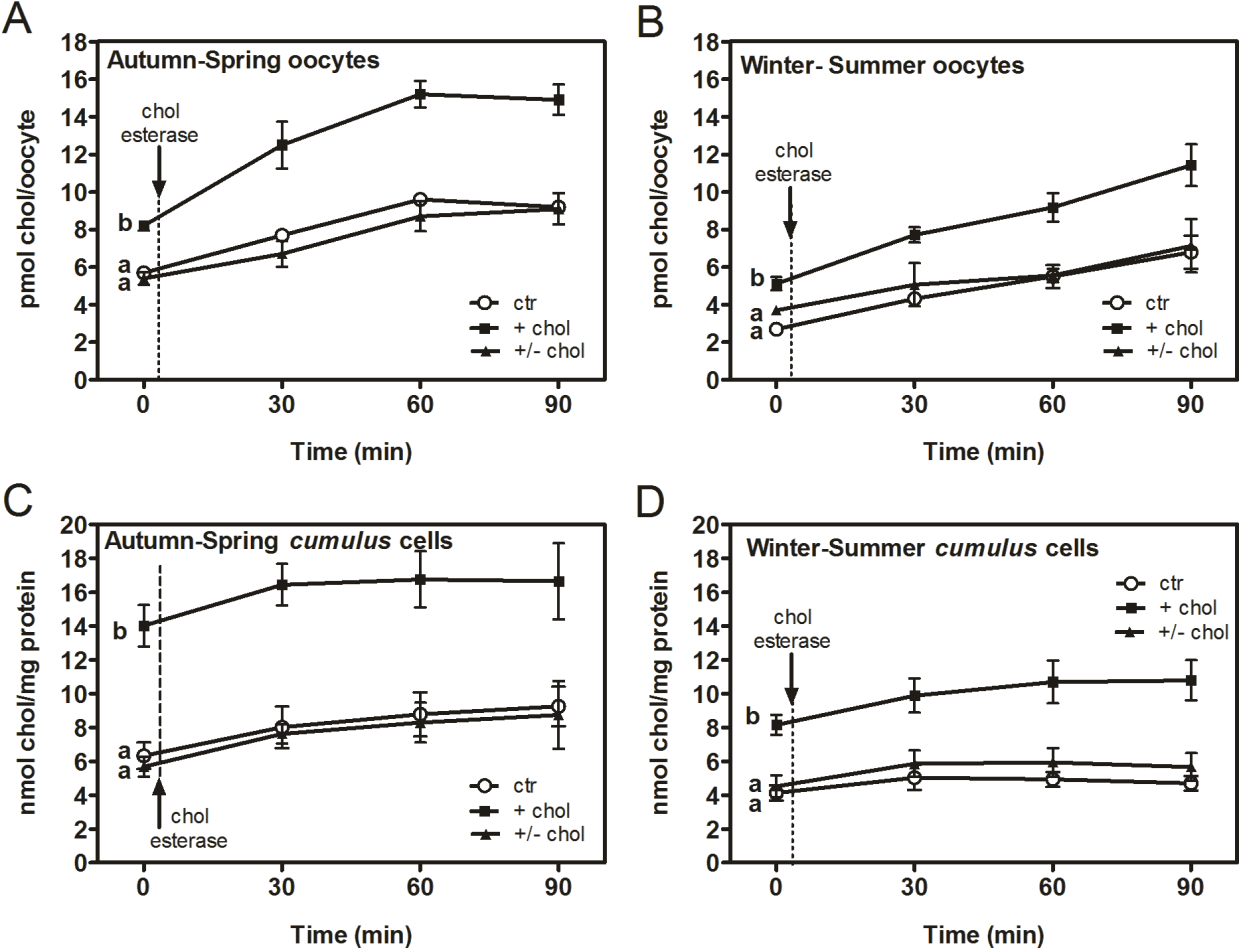
Free cholesterol, cholesterol esters and total cholesterol determined by Amplex® Red in oocytes and *cumulus* cells from autumn and spring compared to those from winter and summer. Free cholesterol and total cholesterol (free cholesterol plus cholesterol esters) levels were measured through a fluorometric method in oocytes and *cumulus* cells from autumn-spring (A,C) and winter-summer (B,D). Total cholesterol was determined adding cholesterol esterase after free cholesterol measurement at time zero. Values are mean ± SEM of 8 replicates for autumn-spring and 8 replicates for winter-summer with n = 45–50 COCs/condition (ctr, +chol, +/-chol), total experiments N = 16. Comparison of means was performed using Bonferroni test for autumn-spring samples and LSD Fisher for winter-summer samples. Different letters (a-b) denote significant differences (*p*<0.05) among treatments within each seasonal period/cellular type. ctr: control; +chol: cells loaded with cholesterol for 45 minutes; +/-chol: cells loaded with cholesterol for 45 minutes and subsequently depleted of cholesterol for another 45 minutes.

To quantify cholesterol incorporation by oocytes and *cumulus* cells, we used a highly sensitive fluorometric method that detects both free cholesterol (membrane cholesterol) and cholesterol esters (stored in LD) using an enzyme coupled reaction. After free cholesterol is measured (time zero), the cholesterol fraction in the form of cholesterol esters is determined by adding a cholesterol esterase within the samples. Measurements were performed in fresh oocytes and *cumulus* cells from untreated COCs, from COCs incubated with MβCD/cholesterol complexes for 45 minutes, and from COCs firstly loaded with cholesterol for 45 minutes and subsequently depleted of cholesterol with empty MβCD for another 45 minutes (Fig 4). An interesting finding revealed that the content of membrane cholesterol in bovine oocytes varies among seasonal periods. Cholesterol was therefore quantified all throughout the seasons. Membrane cholesterol level of oocytes from autumn was observed to be the same as that of spring oocytes, whereas winter oocytes showed a membrane cholesterol level similar to that of summer oocytes (Table 1). Likewise, a difference of 3 pmol of membrane cholesterol per oocyte was found between autumn-spring and winter-summer oocytes, the latter showing the lowest cholesterol level. No differences were found in basal membrane cholesterol of *cumulus* cells among seasons (Table 1). However, when analyzed grouped in both seasonal periods, autumn-spring *cumulus* cells showed a higher membrane cholesterol level than winter-summer cells (p = 0.04). Therefore, cholesterol incorporation and recovery to its original level was analyzed with respect to controls in autumn-spring oocytes versus winter-summer ones (Fig 4A and 4B) and the same criterion was used for the corresponding *cumulus* cells (Fig 4C and 4D).

**Table 1:** Free cholesterol level of bovine oocytes and *Cumulus* cells measured during seasons.

	Free cholesterol			
Seasons	Oocyte		*Cumulus* cells	
	(pmol chol/oocyte)		(nmol chol/μg protein)	
**Autumn**	**5.55 ± 0.22**	**a**	**5.65 ± 1.27**	**a**
**Winter**	**2.41 ± 0.13**	**b**	**3.95 ± 0.25**	**a**
**Spring**	**5.95 ± 0.44**	**a**	**6.84 ± 0.96**	**a**
**Summer**	**3.27 ± 0.70**	**b**	**4.48 ± 1.20**	**a**

Cholesterol levels were measured through a fluorometric method (Amplex Red Cholesterol Assay Kit, Molecular Probes®). Values are mean ± SEM of 4 replicates for each season with n = 45–50 COCs/replicate. Comparison of means was performed using Bonferroni test. Different letters (a-b) denote significant differences (p<0.05) among seasons within each cellular group. chol: cholesterol.

After incubation with MβCD/cholesterol, oocytes showed an increase of membrane cholesterol of ~2.5 pmol/oocyte, regardless of the season (Fig 4A and 4B). C*umulus* cells showed ~2-fold more membrane cholesterol than controls in both seasonal groups (Fig 4C and 4D). Recovery of original cholesterol levels after incubation of cholesterol-loaded COCs with 4.25 mM MβCD was achieved for both oocytes and *cumulus* cells regardless of the season (Fig 4).

Cholesterol esterase hydrolyzed cholesterol esters stored in cytoplasmic LD of autumn-spring oocytes until reaching a *plateau* after 60 minutes of incubation with the enzyme (Fig 4A). This behavior, which was observed under all conditions analyzed, represents -in terms of cholesterol content- total oocyte cholesterol (free cholesterol + esterified cholesterol). As to winter-summer oocytes, hydrolysis of cholesterol esters reached the maximum level after 90 minutes of enzymatic incubation (Fig 4B). Under control conditions, cholesterol esters represented 38% of the total cholesterol content of autumn-spring oocytes while they represented ~60% of the total cholesterol content of winter-summer oocytes (Table 2). In absolute terms, oocyte cholesterol esters remained unchanged among seasonal periods. In *cumulus* cells, ~30% of total cholesterol corresponded to the ester fraction in autumn-spring but accounted only for 19% in winter-summer (Table 2). Hydrolysis of cholesterol esters in *cumulus* cells was more evident during the first 30 minutes of incubation with the enzyme for both seasonal groups (Fig 4C and 4D).

**Table 2:** Fraction of cholesterol esters determined in bovine oocytes and *Cumulus* Cells from different seasonal periods.

	Cholesterol esters							
Seasons	Oocyte		% of Total		*Cumulus* cells		% of Total	
	(pmol chol/oocyte)		Cholesterol		(nmol chol/μg protein)		Cholesterol	
**Autumn-Spring**	**3.53 ± 0.82**	**ns**	**38.19 ± 5.40**		**2.91 ± 0.68**		**31.51 ± 5.63**	
**Winter-Summer**	**4.10 ± 0.69**	**ns**	**60.30 ± 3.41**	*	**0.93 ± 0.31**	*	**19.64 ± 2.49**	*

Cholesterol levels were measured through a fluorometric method (Amplex Red Cholesterol Assay Kit, Molecular Probes®). The fraction of cholesterol that is in the form of cholesterol esters was determined adding cholesterol esterase after free cholesterol measurement and 90 minutes of incubation with the enzyme. Values are mean ± SEM of 8 replicates for autumn-spring and 8 replicates for winter-summer with n = 45–50 COCs/replicate. Asterisk (*) indicates significant differences (p<0.05) between autumn-spring and winter-summer within each cellular group. chol: cholesterol.

### Effect of vitrification on raft marker lipid GM1

**Fig 5:**
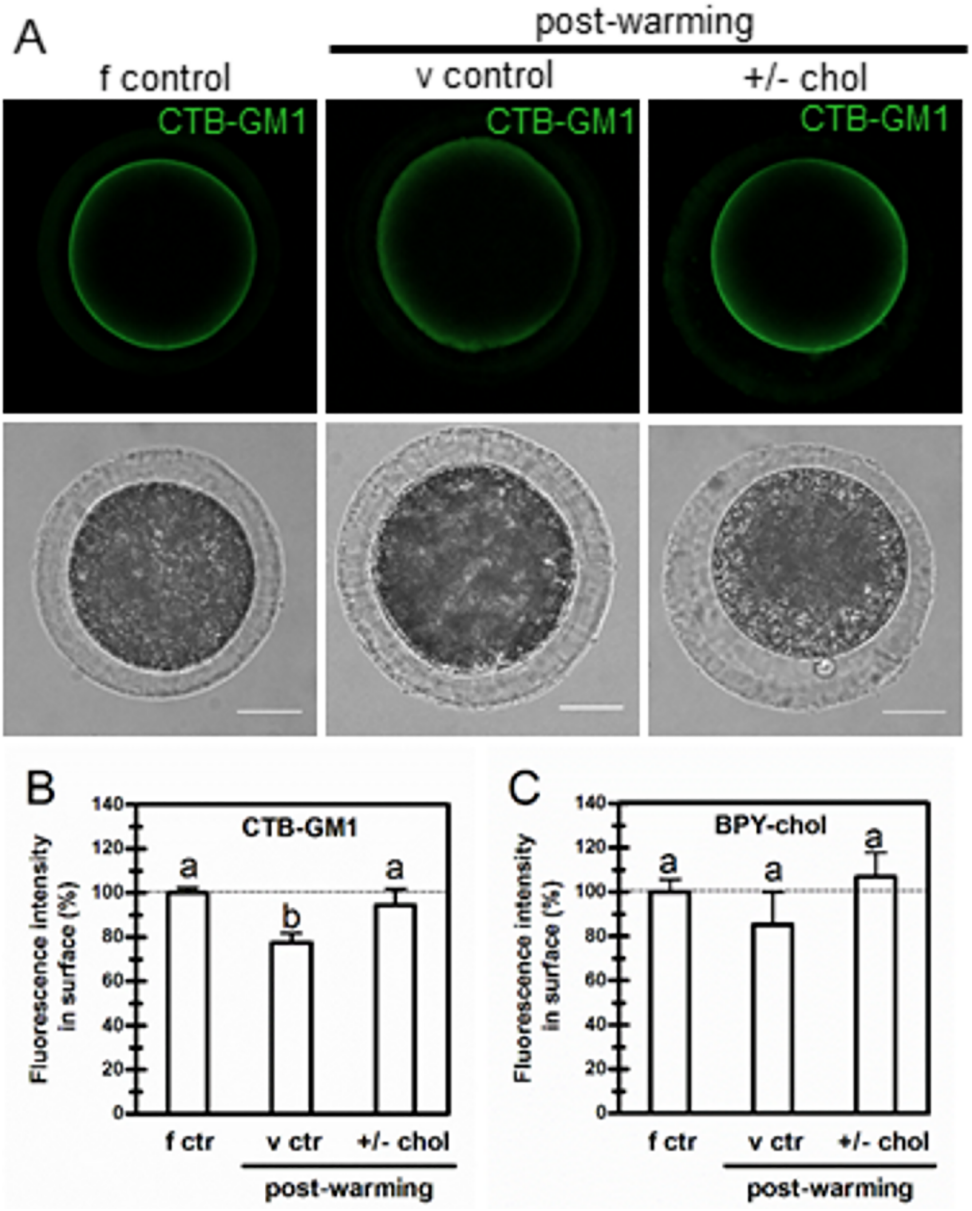
Effect of vitrification on the raft marker lipid GM1. (A) Control and cholesterol-loaded COCs were vitrified with the surface device Cryotech®. After warming, cholesterol-loaded oocytes were exposed to empty MβCD during the recovery period. GM1 was detected in living *cumulus*-free oocytes by using the fluorescent-labeled cholera toxin B subunit (upper panels). Lower panels show bright field; scale bar: 25μm (B) Fluorescence intensity of cholera toxin B subunit-GM1 binding (CTB-GM1) in *cumulus*-free oocytes quantified with *ImageJ* software. Bars represent the mean ± SEM of 3 replicates from a total of 32 fresh control oocytes (f ctr), 36 vitrified control oocytes (v ctr) and 39 vitrified cholesterol-loaded/cholesterol-removed oocytes (+/-chol). Comparison of means was performed using Bonferroni test. Different letters (a-b) denote significant differences (*p*<0.05). (C) Fluorescence intensity of BODIPY-cholesterol (BPY-chol) in ZP-free oocytes quantified with *ImageJ* software. Bars represent the mean ± SEM of 3 replicates from a total of 28 fresh control oocytes (f ctr), 26 vitrified control oocytes (v ctr) and 22 vitrified cholesterol-loaded/cholesterol-removed oocytes (+/-chol). Comparison of means was performed by ANOVA.

The relevance of functional membrane rafts in postcryopreserved bovine oocytes has not been fully explored to date. The degree of compromise of raft marker lipid GM1 in vitrification was analyzed using a fluorescent-labeled cholera toxin B subunit which binds specifically to the ganglioside and permits its identification in living cells. Bovine oocytes showed enrichment in membrane rafts evidenced by the presence of the glycolipid GM1 all along the membrane of living oocytes (Fig 5A). As the size of membrane rafts is smaller than the resolution of light microscopy, this uniform distribution of the GM1-associated fluorescence was expected. Fig 5A (upper and middle panel) shows how vitrification affected both localization and level of GM1 at the plasma membrane. The decrease of GM1-associated fluorescence caused by vitrification (Fig 5B) either did not occur or, if it did, it was at least restored when cholesterol was incorporated into oocytes before vitrification and removed after warming, thus indicating that GM1 related-raft integrity is being preserved by cholesterol modulation. On the other hand, under these experimental conditions, membrane cholesterol showed no differences in terms of BPY-chol fluorescence levels between fresh oocytes and postvitrified treated oocytes (Fig 5C). Postvitrified control oocytes showed a tendency to decrease BPY-chol fluorescence at the plasma membrane. However, the measurement of BPY-chol fluorescence after enzyme treatment to remove ZP after warming revealed high variability and therefore no statistical differences could be observed among the experimental groups. Membrane cholesterol was nonetheless maintained after warming by previously removing the added cholesterol with a concentration of empty MβCD but quite lower (4.25 mM) than that used to enrich membrane cholesterol (15 mM).

## Discussion

Biological membranes undergo a change from a fluid state to an ordered state as temperature is reduced below the transition temperature of the membrane (~10°C in the mature bovine oocyte) [34]. This phenomenon is dependent on their lipid composition. Membranes with high cholesterol:phospholipid ratio or either with polyunsaturated fatty acids or short chain fatty acids are less sensitive to sub-physiological temperatures. Bovine oocytes are particularly susceptible to chilling which occurs at different cellular levels, such as the *zona pellucida*, plasma membrane, meiotic spindles and cytoskeleton [2]. Cholesterol is the major non-polar lipid of mammalian cell membranes, where it typically accounts for 20–25% of total membrane lipid content [35]. The planar structure of cholesterol confers special biophysical properties that contribute to generate a semipermeable barrier and to regulate membrane fluidity [35,36]. Exposing cells to MβCD/cholesterol complexes containing saturating amounts of cholesterol leads to cholesterol enrichment depending on the concentration, exposure duration and cell type [12]. Exposure times to “empty” MβCD required to remove cellular cholesterol are generally much shorter than those needed to enrich a membrane that already has its original cholesterol level. To our knowledge, two studies have been conducted in bovine oocytes using MβCD as a strategy to load cholesterol in their membranes prior to vitrification. In the first study [14], the authors showed that cholesterol diffused through *cumulus* cells and reached the oocyte but failed to reflect these effects in the production of embryos. The same methodology was applied later in immature bovine oocytes [15]. In this case, both the presence of serum and the compact structure of the *cumulus* of immature oocytes could have limited the availability of MβCD/cholesterol complexes, affecting the results collected. Overall, the low success reported in these studies [14,15] could be due to two main reasons which, to our knowledge, have not been taken into account to date. (1) Incubations with MβCD/cholesterol complexes were performed for 25–60 minutes at the concentrations typically used for sperm (0.5–2 mM MβCD) [13]. The lipid mass representing the plasma membrane of an oocyte (diameter ~80–100 μm), which also has numerous *microvilli*, compared to a cell as a sperm, is significantly higher. Therefore, the concentrations and/or incubation times assayed could not have been sufficient to effectively increase the level of cholesterol in the membranes of oocytes. (2) If before vitrification, there was a genuine incorporation of cholesterol into the oocyte plasma membrane, after warming it would be necessary to remove the added cholesterol to recover the physiological level of cholesterol at the membrane. In the cholesterol-loaded sperm, this scenario seems not to affect them to a great extent since cryopreservation itself produces an efflux of cholesterol that may recover -either totally or partially- cholesterol levels in the membrane after thawing (this being the reason why it is said that sperm undergo "cryocapacitation"). In the present work, we have increased MβCD/cholesterol concentration and we have analyzed two incubation times (45 minutes and 2 hours). We have also hypothesized that removal of cholesterol after warming better preserves the organization of the plasma membrane by recovering its original cholesterol level.

*In situ* detection of activated caspases revealed that modulation of cholesterol for 45 minutes does not affect oocyte apoptotic status. On the contrary, incubation for 2 hours increases apoptosis after vitrification. Based on this, it can by hypothesized that the level of apoptosis of oocytes loaded with cholesterol for 45 minutes, vitrified and recovered (cholesterol removed), could be due at least by the cytotoxicity caused by cryoprotectants since no differences were found with respect to the toxicity control group. However, no significant differences were found either between the apoptotic level of these oocytes and the fresh controls. It was, in fact, the only experimental group that showed this condition compared to fresh oocytes, thus indicating a level of cell death closer to that of fresh oocytes. Interestingly, it has been shown that cholesterol protects the phospholipid bilayer from oxidative damage [37]. Also, most part of *cumulus* cells was found to be positive for caspases and propidium iodide, a marker of cell viability that penetrates when the membrane loses integrity. Vitrification protocols are not particularly designed to preserve *cumulus* cells but the oocyte, with the large volume that this cell type presents. This raises a controversy at the moment of vitrifying bovine oocytes with the corona *radiata* and some layers of *cumulus* cells to proceed to the subsequent *in vitro* fertilization (IVF), as is usually done. In mammals, fertilization rates in oocytes without *cumulus* are markedly low [38]. Furthermore, intracytoplasmic sperm injection (ICSI), which is performed with success in human and mouse denuded oocytes (without *cumulus*), still remains inefficient in the bovine [39]. Because vitrified bovine oocytes lack great part of viable *cumulus* cells capable of responding to the signaling cascades generated by sperm, further studies should converge in the adaptation of an IVF protocol for denuded (and vitrified) oocytes. Recent research has demonstrated that the presence of several layers of *cumulus* cells during vitrification reduces the survival of bovine matured oocytes [40]. This research also showed that vitrifying oocytes either with a few layers of *cumulus* cells or without *cumulus* cells, but co-incubated with fresh COCs during fertilization, may lead to higher survival and embryo development.

Our data showed that cholesterol was incorporated into the oocyte plasma membrane as evidenced by comparative labeling of the fluorescent probe BPY-chol. However, direct exposure of ZP-free oocytes to empty MβCD was not sufficient to achieve cholesterol removal in oocytes with physiological levels of cholesterol. Instead, it affected the distribution of oocyte LD which may reflect that cholesterol repletion occurs at the plasma membrane. It seems possible that cholesterol removal occurs concomitantly to cholesterol replenishment driven by cortical LD through specific hydrolysis of cholesterol esters stored within these organelles [41], thus maintaining BPY-chol labeling at the plasma membrane. Macro-autophagy of LD, termed lipophagy, is a physiological mechanism to degrade LD in some circumstances [42]. It is important to note that this effect was only observed in oocytes with physiological levels of cholesterol exposed to MβCD but not in cholesterol-loaded oocytes in which excess cholesterol was fairly simple to remove. Nevertheless, under this experimental condition (ZP-free oocytes exposed to MβCD), restructuring or even disturbance of the underlying cytoskeleton cannot be discarded. At present, LD have gain notoriety due to increasing evidence related to their roles in many aspects of health and disease [43]. The prevalence of metabolic syndromes, obesity, steatosis and atherosclerosis has prompted further biomedical research on LD since early embryo stages or even at the oocyte level [44].

On the other hand, earlier studies have addressed the analysis of the fatty acid composition of total phospholipids, major phospholipid sub-classes and triacylglycerols from bovine oocytes and embryos [5,45–47]. However, none of these studies have quantified oocyte cholesterol levels, except for the work of Kim et al. [45] who measured total cholesterol content. More recently, mass spectrometry has provided new insights into the composition of different molecular species of phospholipids, free fatty acids, and triacylglycerols present in single oocytes and preimplantation embryos [48,49]. Relative abundance of squalene, a key intermediate in cholesterol biosynthesis, showed higher levels in bovine immature oocytes compared to *in vitro* matured oocytes analyzed through this approach [49]. In our work, an analytical strategy has been conducted to quantify free cholesterol and esterified cholesterol in bovine oocytes and *cumulus* cells. An interesting finding revealed that the level of membrane cholesterol in bovine oocytes varies among seasonal periods. In contrast, cholesterol esters remained stable among seasons. These variations at the membrane level were enough to account for the differences also found in total cholesterol. Even when no design was performed to detect a direct effect of the season, in particular, these results could be quite close to show this effect. For subsequent analysis, oocytes were grouped into autumn-spring and winter-summer oocytes taking into account similar levels found in membrane cholesterol among seasons. Autumn and spring oocytes evidenced the highest content of membrane cholesterol as well as of total cholesterol. Equal amount of total cholesterol was found by Kim and co-workers [45] in bovine oocytes matured in a serum-free medium (9.2 pmol per oocyte), irrespective of the season. However, when oocytes were matured in the presence of fetal bovine serum, cholesterol content was significantly higher (15.1 pmol per oocyte) which can be explained by the fact that serum provides a variety of lipids including cholesterol [50]. On the other hand, our results showed that the differences found in total cholesterol of *cumulus* cells were explained by the differences found in the content of membrane cholesterol and of cholesterol esters. Furthermore, membrane cholesterol of *cumulus* cells showed the same pattern as that of oocytes when grouped in these seasonal categories. Our results demonstrate that the whole COC is dynamic in terms of cholesterol homeostasis.

In addition, both oocytes and *cumulus* cells increased membrane cholesterol after incubation with MβCD/cholesterol and recovered the original level, regardless of the season. Also, total cholesterol was restored to physiological levels. Interestingly, cholesterol-loaded oocytes from winter and summer showed that membrane cholesterol levels were the same to those of autumn-spring oocytes at basal conditions. Similar profiles were also observed in the cholesterol ester fraction and total cholesterol. Altogether, these results suggest a distinctive cholesterol metabolic status of COCs among seasons. In dairy cows, a seasonal study showed a higher percentage of saturated fatty acids in phospholipids of germinal vesicle oocytes and granulosa cells during summer whereas mainly mono- and polyunsaturated fatty acids (PUFA) predominated during winter [5]. Biophysical analysis of oocyte membranes revealed that the lipid phase transition decreased 6°C between summer and winter [5]. These alterations in membrane composition were associated with decreased developmental competence during high ambient temperature months. Conditions of heat stress may aggravate the negative energy balance of dairy cows contributing to the metabolic imbalance responsible for reduced fertility [51]. Heat stress seems, in fact, to be relevant for reproductive performance of cows in tropical and subtropical climates. In this respect, *in vivo* produced embryos of *Bos indicus* during the rainy season, compared to those produced in the dry season, had a lower number of apoptotic cells in fresh embryos as well as after freezing [52]. A connection between nutrition and quality of oocytes and embryos has been suggested [51,53,54]. Nevertheless, even when there is some coincidence in that PUFA rich diets have beneficial effects on bovine oocyte and embryo quality [55–59] correlation to changes in gamete lipid composition still remains to be established. It has also been proposed that PUFA uptake by the oocyte is selective and highly regulated to avoid the risk of cellular damage [57,58,60]. A recent study has shown that dietary PUFA supplementation has no effect on the relative abundance of phosphatidylcholine or sphingomyelin molecular species in bovine blastocysts produced *in vitro* [61]. As to cholesterol, it is known that plants only have minor amounts of cholesterol [62] and that the diet is therefore not a cholesterol source for herbivorous, however, a particular nutritional condition may influence the metabolic status of oocytes and embryos. Cholesterol may account for up to 45 mol% of the membrane´s total lipid content in animal cells [63]. Measuring only choline-phospholipids and total cholesterol, Kim et al. [45] showed that cholesterol to phospholipid ratio is important in bovine oocytes. Our work provides new data on unesterified cholesterol and cholesterol esters in bovine oocytes and *cumulus* cells at different seasonal periods.

Taking into account that cryopreservation may disturb plasma membrane organization required for fertilization, we analyzed putative raft molecule GM1 in living oocytes. Gangliosides have been found to be involved in multiple physiological functions, and it is important to understand how their distribution is regulated in the cell membrane [64]. It has been shown that the clustering of both GM1 and GM3 at the plasma membrane depends primarily on membrane cholesterol levels and also on intact actin cytoskeleton [64]. To our knowledge, this is the first report in which ganglioside GM1 is described in the bovine oocyte. Vitrification was observed to clearly affect the localization and level of GM1 at the plasma membrane. In line with this, dispersion of GM1 clusters by chilling was reported in mouse fibroblast analyzed by immunoelectron microscopy after quick-freezing and freeze-fracture [65]. However, when cholesterol was incorporated into oocytes before vitrification and removed after warming, GM1 levels at the plasma membrane were not altered or at least restored. Likewise, membrane cholesterol was maintained after warming. Cholesterol modulation during vitrification and warming could therefore be a useful tool to preserve membrane raft integrity. A glycosylphosphatidylinositol (GPI)-anchored protein named Juno has been identified as the sperm receptor at the plasma membrane of mammalian oocytes [66]. GPI-anchored proteins are enriched in non-invaginated membrane rafts highlighting how important plasma membrane organization is for fertilization. Further analyses will be needed to address membrane lipid compromise during vitrification of bovine oocytes. Emerging studies in embryos have found differences between fresh and vitrified bovine blastocysts in certain molecular species of choline-phospholipids and triacylglycerols [61,67].

Cholesterol is a key contributory factor to membrane properties, particularly membrane fluidity. This is the first study conducted to date on free cholesterol and cholesterol esters in bovine oocytes and *cumulus* cells. Interestingly, cholesterol levels differed among seasonal periods revealing a distinctive metabolic status of COCs. Also, the whole COC showed a dynamic organizational structure in terms of cholesterol homeostasis. Modulation of membrane cholesterol by MβCD improved oocyte survival after vitrification yielding levels of cell death closer to those of fresh oocytes. Oocytes effectively incorporated membrane cholesterol prior to vitrification and recovered their original level after cholesterol removal. Cholesterol modulation also preserved membrane localization of the raft lipid GM1 after vitrification, thus suggesting its possible role as a cryotolerance marker. Future studies on the biophysical properties of oocyte and *cumulus* cell membranes will provide new insights to understand the role of lipid behavior in the developmental competence of cryopreserved oocytes.
